# Perceptions of professional nurses regarding the National Core Standards tool in tertiary hospitals in KwaZulu-Natal

**DOI:** 10.4102/curationis.v43i1.1971

**Published:** 2020-04-01

**Authors:** Winnie T. Maphumulo, Busisiwe R. Bhengu

**Affiliations:** 1 Department of Health, Faculty of Nursing, University of KwaZulu-Natal, Durban, South Africa

**Keywords:** NCS, perceptions, professional nurses, tertiary hospitals, quality

## Abstract

**Background:**

Internationally, healthcare providers share a common goal of providing safe and high-quality care to every patient. In South Africa, the National Core Standards (NCS) tool was introduced to improve the quality of healthcare delivery.

**Objectives:**

This article is aimed to determine the perceptions of nurses concerning the use of NCS as a tool to measure quality care delivery in tertiary hospitals in KwaZulu-Natal.

**Method:**

This was a cross-sectional descriptive survey, where a purposive sampling technique was used to select hospitals. Six strata of departments were selected using simple stratified sampling. In each stratum, every second ward was selected from the provided list of wards using a systematic random sampling. The population of professional nurses in selected departments was 3050, from which 437 participants were selected by systematic random sampling. The collected data were analysed using Statistical Package for the Social Sciences (SPSS^®^) version 25.

**Results:**

The study indicated that 53.5% respondents believed that the NCS tool allows them to identify areas of weakness, pointing to risks in basic human rights. However, only 49.7% respondents believed that the NCS tool allows staff inputs to identify relevant innovations. The study recommends improvement in the organisational climate and adoption of strategies that add value to patient care.

**Conclusion:**

Professional nurses perceived the NCS tool as a good tool for improving quality of healthcare delivery, but there is a need to improve environmental practice and involvement of all healthcare establishments to increase its effectiveness.

## Introduction

Globally, the healthcare providers share a common goal of providing safe and high-quality care to every patient all the time (Babiker et al. 2014:10). The government established the Office of Health Standards Compliance (OHSC) to fulfill the constitutional obligation of ensuring the delivery of safe and high-quality care in health establishments (National Department of Health (NDoH) 2013:8). The OHSC introduced a quality assurance mechanism to regulate the quality of health services against a prescribed set of norms and standards prescribed in the *National Health Amendment Act* (Act No. 12 of 2013) (NDoH 2013:8). The OHSC developed the National Core Standards (NCS) tool, which serves as a guide for managers at all levels, explaining the expected level of service delivery (NDoH 2013:23). The NCS tool provides the minimum standards of care that are mandatory in all health establishments in South Africa (Ranchod et al. 2017:106). The main aim of NCS is to develop a common definition of quality care and to launch a benchmark against which healthcare organisations could be assessed (NDoH 2013:17).

Commonly, professional nurses constitute a larger part of healthcare industry. Therefore, their perceptions of the NCS tool are essential because they can influence the implementation of the tool either positively or negatively. Professional nurses are also gatekeepers of quality care delivery and often have a role of coordinators of a multidisciplinary care. The purpose of the study was to assess the perceptions of nurses concerning the use of NCS as a tool to improve quality care delivery. Success in the implementation of any quality initiative in a healthcare organisation is determined by its acceptability by the largest possible number of employees in that organisation (Boonstra, Versluis & Vos 2014:370). Gaps in the interpretation of the NCS tool could indicate a need for education to empower users regarding their roles and responsibilities. This article reports on part 3 of the author`s main study called ‘Analysing the process of implementation of the National Core Standards as a tool for ensuring quality care delivery in tertiary hospitals in KwaZulu-Natal’.

## Background

Concerns about the quality of healthcare delivery and performance improvement are driving significant changes in healthcare systems globally (Whittaker et al. 2011:60). However, it has to be acknowledged that the term ‘quality in healthcare’ is a subjective and multifaceted concept, which many authors define in different ways, and these definitions vary amongst countries and stakeholders and over time (Hakeem & Thanikachalam 2014:415). No single definition of quality in healthcare services applies in all situations, and understanding the definition of quality requires many different measures (Nylenna et al. 2015:3). The Department of Health in South Africa defines quality as the ability to attain the best possible health outcomes using the available resources (NDoH 2013:16). The literature related to the meaning, definition and perception of nurses about quality nursing care is limited (Burhans & Alligood 2010:10). According to the Burhans and Alligood’s (2010:21) study, nurses defined the quality of nursing care as related to six vital themes, such as empathy, caring, responsibility, intention, respect and advocacy. However, the nursing literature often uses ‘Donabedian’s definition of quality’ and ‘Donabedian’s model’ (Kelley et al. 2011:155). Donabedian’s framework, the Lean system (Poksinska 2010:319). and the NCS tool formed the conceptual framework of this study.

## Conceptual framework of the study

For this study, a questionnaire was developed using the Donabedian variables, Lean principles and the seven domains of NCS to determine the perceptions of professional nurses regarding the ability of the NCS tool to improve the quality of healthcare delivery.

Donabedian’s definition of quality care embodies the entire range of variables from structures to processes to health outcomes (Nocella et al. 2016:20). *Structure* denotes the features of the setting in which care takes place. Structure measures system inputs such as human resources, infrastructure, availability of equipment and supplies, including operational tools such as policies and protocols (Nocella et al. 2016:20).

*Process* measures what is really carried out when providing care. It addresses activities or interventions carried out within an organisation for the care of patients, such as patient education, training, promotion of teamwork, patient care activities, equipment maintenance and so on (Nocella et al. 2016:20). Donabedian understood that organisational structure has an impact on the ability of healthcare organisations to successfully implement and sustain quality improvement initiatives if well-designed systems or processes are implemented. Outcomes refer to the effect of intervention (e.g. an improvement in quality healthcare delivery) subsequent to the health services received. This includes intended outcomes, such as relief from pain, and unintended outcomes, such as complications (Nocella et al. 2016:20). According to Donabedian, good structure increases the likelihood of good process, and good process increases the likelihood of good outcome. His work led to an understanding of the system’s approach in evaluating health establishments (Halasa et al. 2015:98).

Another push for continuous improvement is the need for removal of waste from available resources and concentrating on value-added processes whilst respecting the employees as recommended in the Lean system (Sisson & Elshennawy 2015:263). This was fueled by the increasing costs of healthcare (Moraros, Lemstra & Nwankwo 2016:151). According to Poksinska (2010), Lean principles are as follows: to determine value from a client’s standpoint, define the value stream, maintain a continuous flow, pull production, integrate the supply chain, focus on quality, visual management, use technology that serves employees and processes, human resource development and continuous improvement.

The NCS tool addresses crucial issues that are vital for providing quality care (NDoH 2011:7). In order to attend to life-threatening issues in quality delivery and patient safety, the NCS tool was structured into seven domains (NDoH 2011:6). According to the World Health Organization (WHO, 2006:6), a domain is a part of service delivery where safety or quality could be jeopardised. Table 1 shows the NCS tool as tabulated by NDoH.

**TABLE 1:** The National Core Standards’ domains.

Domain	Scope
Patient rights	This domain sets out what a healthcare establishment must do to make sure that patients are respected and their rights are upheld in accordance with the Batho Pele principles and the Patient Rights Charter (NDoH 2011:17).
Patient safety, clinical governance and clinical care	This domain emphasises the need for clinical governance to ensure quality care and ethical practice. It aims to mitigate adverse events, including healthcare-associated infections, and supports any infected patient and staff (NDoH 2011:22).
Clinical support services	This domain stresses specific systems and services essential to develop, monitor and maintain efficient patient care, including timeous availability of medicines and provision of effective diagnostic and therapeutic medical equipment (NDoH 2011:26).
Public health	This domain explains how integrated quality care is provided for the whole community, healthcare organisations co-operating with non-government organisations (NGOs), local communities and other relevant healthcare providers in relevant sectors to promote health, prevent illness, reduce further complications and prepare for disaster (NDoH 2011:30).
Leadership and governance	This domain covers the strategic functions of communication, public relations, oversight, accountability, risk management, quality management and leadership. It encompasses proactive leadership offered by senior management through effective planning and risk management supported by hospital board, clinic committee and the relevant supervisory structures (NDoH 2011:34).
Operational management	The domain covers the day-to-day responsibilities involved in supporting and ensuring delivery of safe and effective patient care, including management of human resources, finances, assets and consumables, and information and records (NDoH 2011:38).
Facilities and infrastructure	This domain covers the requirements for clean, safe and secure physical infrastructure such as buildings and equipment, and stresses the need for effective waste management, availability of linen and laundry services (NDoH 2011:43).

*Source*: National Department of Health (NDoH), 2011, *National core standards for health establishments in South Africa:`Towards quality care for patients’*, Department of Health, Republic of South Africa, Tshwane

### Operational definitions of different terms

*Perception*: It is the act of noticing or being aware or a comprehension or an understanding of something, and interpreting it from the external world by means of sensory receptors (Pickens 2005:53). In the study, perceptions mean understanding or comprehension of professional nurses, unit managers and nurse managers regarding the NCS tool.

*Tertiary hospital*: A hospital that provides a highly specialised consultative healthcare service for inpatients and outpatients on referral basis from a primary or secondary healthcare service. It has advanced expertise and technical equipment for advanced medical investigations and treatments National Department of Health (NDoH 2013:2).

*Professional nurse*: A person who has completed a 3- or 4-year diploma or 4-year degree course and is registered under the *Nursing Act* No. 50 of 1978 and renders comprehensive nursing care independently in clinical area (Republic of South Africa 2005:25). In this study, professional nurse means a nurse who has a basic diploma or degree in nursing, including a nurse having a postgraduate diploma and degree in nursing.

## Problem statement

South African Medical Association (SAMA, 2015:40) contends that government tools and frameworks are usually implemented poorly because they are often impractical and look ‘good’ only on paper. South African Medical Association (2015:42) also states that it is impossible to expect that the NCS tool will dramatically improve the quality of healthcare delivery in the under-resourced environment because of lack of financial and human resources accompanied by mishandling of funds by government officials. This literature may negatively influence the perceptions of nursing practitioners who carry most of the burden of quality improvement and innovations.

The principal investigator, with over 10 years of experience working with the NCS tool, believes that this is a good tool. However, she aligns herself with SAMA’s argument, as she has also experienced the impracticability of the tool in real situations related to shortage of staff and equipment. Moreover, the users of the tool around KwaZulu-Natal seem to be unclear and frequently mourn about its implementation. This could mean that there is a problem of perception of NCS tool amongst its users.

The study was carried out in KwaZulu-Natal because between 2008 and 2015, there was a drastic rise in medico-legal claims (Pieterse & Erasmus 2017); the province still has an amount of over R5 billion as pending claims. It was also convenient for the researcher to conduct thorough study because she was based in KwaZulu-Natal.

## Research methodology

### Study design

A cross-sectional descriptive survey was carried out amongst professional nurses in selected hospitals from 15 January 2017 to 30 May 2017. The researcher made appointments with nurse managers and assistant nurse managers in each of the four hospitals selected to explain the nature and purpose of the study. Delays were experienced whilst trying to have appointments with management, and further delays occurred in some hospitals whilst waiting to have suitable dates for data collection. Data were collected intermittently because of the different dates provided by different departments of different hospitals for data collection.

### Study purpose

The aim of this study was to assess the perceptions of nurses concerning the use of NCS as a tool to measure quality care delivery in KwaZulu-Natal.

### Research site

This study was conducted in four hospitals that offer tertiary services in KwaZulu-Natal, South Africa. KwaZulu-Natal province was chosen because of its diversity amongst the South African provinces in terms of languages, culture and provincial legislature, which can interpret and implement national policies differently.

Two tertiary hospitals (A and B) situated in the eThekwini district provide both secondary and tertiary services. The third tertiary hospital (C) is located in Pietermaritzburg in the Msunduzi district serving the western half of KwaZulu-Natal, which includes the following districts: uMgungundlovu, uThukela, uMzinyathi, Amajuba and Harry Gwala. The fourth hospital (D) is situated in Empangeni in the uMkhanyakude district, serving uThungulu, uMkhanyakude and Zululand health districts.

### Study population

The target population for the study included 3050 professional nurses employed on a full-time basis in four selected hospitals. Professional nurses are leaders in the implementation of the NCS tool, so their perspectives and experience could provide important information to improve tool’s quality. Hence, their positive engagement in the implementation of quality improvement programmes could lead to positive health outcomes.

### Eligibility criteria

*Inclusion criteria:* All professional nurses having more than 2 years of experience in the field, permanently employed in these selected hospitals, willing to participate and available during the study period were included in the study. Both day and night nurses were considered.

*Exclusion criteria:* Professional nurses who were off duty or on leave (vacation, maternity, sick or study leave) during data collection were excluded. All professional nurses who were having management positions of all levels as well as who have less than 2 years of experience in the field were also excluded from the study.

### Sampling technique

Purposive sampling was used to select four hospitals offering tertiary services in the province. These were further stratified into six strata by using simple stratified sampling, namely, stratum 1: medical, stratum 2: surgical, stratum 3: critical care unit, stratum 4: high care, stratum 5: paediatrics and stratum 6: obstetrics. In each stratum, every second ward was selected from the list of wards provided by the nurse manager using systematic random sampling. Convenience sampling was used to select professional nurses from both day and night shifts in these selected hospitals. A total sample size of 437 respondents was used. The number of respondents in each stratum was as follows: stratum 1: medical = 125, stratum 2: surgical = 95, stratum 3: critical care unit = 127, stratum 4: high care = 17, stratum 5: paediatrics = 60 and stratum 6: obstetrics = 13.

### Sample size

**FIGURE 1:**
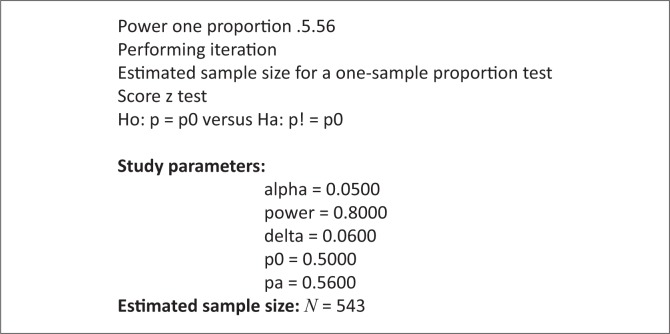
Sample size calculated using Stata V13 statistical software.

A sample size of 543 respondents (±6%) was required to estimate the correct proportion of professional nurses’ perceptions of the NCS tool. This number provided a 95% probability of achieving the study’s objectives and assumed that 50% would yield a clear picture of their perceptions of the tool. The sample size was computed by using the Stata V13 statistical software. The command was power one proportion. Figure 1 shows the sample calculated using Stata V13 statistical software.

### Instrument for data collection

A closed-ended questionnaire was used for collecting data. This questionnaire’s design was based on the researcher’s use of specific items of the NCS tool, Donabedian framework and the Lean system, which were modified to suit the purpose of this study.

#### Data collection technique

Although the time taken to complete each questionnaire was approximately 20 min, the researcher had to personally obtain consent, hand out questionnaires, explain them and collect the data. This was carried out during the nurses’ available time, for example, during tea or lunch breaks or on rare occasions during a ward meeting arranged by management for day and night nurses. After every meeting, respondents deposited their completed questionnaire in the enclosed box provided in the duty room. Participants were provided enough time to complete the questionnaire at their will if they desired so. For the latter participants, the questionnaires deposited in the box were collected by the researcher approximately 1 week after the initial meeting.

#### Scientific rigour

The questionnaire was tested and validated to ensure understanding and meaning of the presented concepts and simplicity of statements, and to determine the time taken for completing it during the pilot testing. The respondents used in the pilot study were marked by using wards not used in the main study to enable them to be excluded from the main population. Readability and comprehension were verified by the supervisor having research background and two quality managers from two participating hospitals in the eThekwini district. A content validity was also performed, whereby the items of the research instrument were compared with the objectives of the study to ensure that the tool was measuring what it purported to measure. The degree of correlation between items on a scale was validated using Cronbach’s alpha coefficient. The questionnaire had a good internal consistency with a Cronbach’s alpha coefficient of 0.851. Pilot data did not lead to the modification of materials or procedures.

### Data analysis

Data were analysed using the Statistical Package for the Social Sciences (SPSS^®^) version 25 software. A structured questionnaire was used to measure professional nurses’ perceptions for the use of the NCS tool using the Likert scale. Data on 5-point Likert scale were coded as follows: 1 = strongly disagree, 2 = disagree, 3 = neutral, 4 = agree and 5 = strongly agree. The Likert scale data were recoded to agree (strongly agree and agree), disagree (disagree and strongly disagree) and neutral (neutral and missed). Data were reported on both demographic data and perceptions of professional nurses about three features of quality interventions as described by Donabedian, namely, structure, processes and outcomes.

## Ethical consideration

Ethical approval was obtained from the Humanities Research Ethics Committee of the University of KwaZulu-Natal (HSS/1905/016). Permission was obtained from the KwaZulu-Natal Department of Health and the managers of participating institutions and departments. Written consents were obtained from the respondents after explaining the research study, including potential risks and their mitigation. Risks would include disruption in ward activities and relaxation time, and these were mitigated by giving them questionnaires to be completed during break periods. The respondents requiring longer time to complete questionnaires were allowed to take them along and asked to deposit completed questionnaires within a week in the sealed box provided in the unit. Participation in the study was voluntary. Confidentiality and anonymity of the respondents were maintained throughout the study by using assigned codes and numbers to each questionnaire so that it was not possible to link questionnaire with individual respondent.

## Results

Although the minimum calculated sample size requirement was 543 participants, a total of 500 professional nurses available in the wards were approached for participation in the study. About 466 questionnaires were returned, yielding a response rate of 93.2%. After discarding 29 questionnaires for non-adherence to instructions, the final sample included 437 questionnaires (87.4%). Table 2 shows the strata-wise distribution of respondents.

**TABLE 2:** Distribution of respondents.

Strata	Number of respondents
Medical	125
Surgical	95
Critical Care	127
High Care	17
Paediatrics	60
Obstetrics	13
**Total**	**437**

### Socio-demographic characteristics of respondents

This study included only full-time employed professional nurses. Respondents were distributed across the institutions as follows: hospital A = 147/437 (33.6%), hospital B = 82/437 (18.8%), hospital C = 108/437 (24.7%) and hospital D = 100/437 (22.9%). The majority of respondents (351/437, 80.3%) were females. Most respondents (224, 51.3%) had a basic diploma in nursing (R425 and R63), and 115 (26.3%) had a specialty diploma in addition to their basic qualifications. The results also showed that most of these respondents (386, 88.3%) had a working experience ranging from 6 years to more than 20 years.

### Perceptions of nurses about the application of structure standards of the National Core Standards tool

Slightly more than half of the respondents (225, 51.4%) felt that the organisational arrangements encouraged them to apply the NCS tool well as they had clear job descriptions. Only 183 (41.8%) respondents believed that the organisational arrangements offered clear lines of communication to enable them to apply the tool well. The lowest score in this section was 169 (38.6%), where respondents felt that the organisational arrangements encouraged them to apply the tool well because they had autonomy in decision-making. Table 3 shows the perceptions of the nurses about the structure standards of the NCS tool.

**TABLE 3:** Nurses’ perceptions about the application of structure standards of the National Core Standards tool.

Perceptions about the application of structure standards	Agree		Neutral		Disagree	
	*n*	%	*n*	%	*n*	%
Organisational arrangements offer clear definition of job description	225	51.4	67	15.3	145	33.1
Organisational arrangements offer clear lines of communication	183	41.8	81	18.5	173	39.5
Organisational arrangements offer autonomy in decision-making	169	38.6	83	19.0	185	42.3

### Perceptions of nurses about the application of process standards of the National Core Standards tool

The highest level of agreement was for ‘adherence to existing standards and guidelines is part of staff performance criteria’, with 252 (57.6%) respondents agreeing with this statement. Most respondents (232, 53%) felt that the standards put into the NCS tool encourage patient-centred care. The lowest level of agreement was for ‘implementation of the NCS and its success is completely dependent on nurses only’, with only 164 (37.5%) respondents agreeing with this statement.

A significant number of respondents (251, 57.4%) believed that the NCS tool emphasised on multidisciplinary approach and the need for harmony at the workplace. The majority of respondents (248, 56.7%) indicated that they believed that monitoring patient satisfaction surveys helped health establishment to determine the needs of customers, and about 247 (56.5%) respondents felt that the NCS tool allowed for the continuity of patient care. About 207 (47.3%) felt that the NCS tool allowed them to practise according to their scope of practice. Less than half of the respondents (193, 44.1%) believed that nurses’ skills were utilised appropriately when implementing the tool.

The highest level of agreement for the elimination of waste processes was for ‘NCS ensures the elimination of waste due to production defects, e.g. medication errors’, with 207 (47.3%) respondents agreeing with this statement. The lowest level of agreement was for ‘NCS ensures the elimination of waste due to excess processing, e.g. ordering more diagnostic tests than the diagnosis warrants’, with 187 (42.7%) respondents agreeing with this statement. Only 206 (47.3%) respondents felt that the NCS tool improved waiting period of patients awaiting treatments, whilst 203 (46.4%) believed that the NCS tool ensures that wastage of time looking for equipment is eliminated.

About 205 (46.9%) respondents felt that the NCS tool ensures that product waste is eliminated when transporting or waiting for surgical sundries to arrive, whilst 202 (46.2%) believed that the NCS tool ensures that the waste in excess processing is reduced. Table 4 shows nurses’ perceptions about process standards of the NCS tool.

**TABLE 4:** Nurses’ perceptions about the application of process standards of the National Core Standards tool.

Variables	Agree		Neutral		Disagree	
	*n*	*%*	*n*	*%*	*n*	*%*
**Perceptions about the application of the process standards**						
Adherence to the existing standards and guidelines is part of the staff performance criteria	252	57.6	41	9.40	144	32.90
The NCS tool emphasises the multidisciplinary approach and harmony at the workplace	251	57.4	51	11.70	135	30.80
Monitoring patient satisfaction surveys helps to determine the needs of customers	248	56.7	40	9.20	149	34.00
NCS allows for the continuity of patient care	247	56.5	52	11.90	138	31.50
NCS enables identification of areas of weakness, e.g., potential risks to patient safety	245	56.0	49	11.20	143	32.70
An independent body should perform accreditation checks to ensure credibility of the findings	239	54.6	50	11.40	148	33.80
Standards put into the NCS tool encourage patient-centred care	232	53.0	48	11.00	157	35.95
The NCS tool allows me to practice according to the scope of practice	207	47.3	61	14.00	169	38.60
Nurses’ skills are utilised when implementing the NCS tool	207	47.3	82	18.80	162	37.00
Implementation of NCS and its success is completely dependent on nurses only	164	37.5	45	10.30	228	52.10
**National Core Standards ensure the elimination of waste because of the following processes**						
Production defects, e.g., medication errors, faulty material	207	47.3	72	16.50	158	36.10
Time wasted (waiting for bed assignment, unnecessary queues)	206	47.3	74	16.90	157	35.90
Waste during transportation, e.g., unnecessary movement of staff and material	205	46.9	85	19.50	147	33.60
Waste during movement, e.g., nurses waste time looking for equipment	203	46.4	73	16.70	161	36.80
Waste because of repetition, e.g., entering repetitive information on multiple documents	202	46.2	85	19.55	150	34.30
Waste of inventory, e.g., poor stock control, keeping patients who could be discharged	199	45.5	56	12.60	182	41.62
Waste in excess processing, e.g., ordering unnecessary diagnostic tests, unnecessary referrals	187	43.0	64	14.60	186	42.50

NCS, National Core Standards.

### Perceptions of nurses regarding outcome standards of the National Core Standards tool

The highest level of agreement for this section was for ‘the NCS promotes the use of feedback to improve service delivery’, with 232 (53%) respondents agreeing with this statement. The lowest level of agreement was for ‘the NCS promotes the mentoring system’, with only 193 (44.1%) of the nurses agreeing with this statement.

More than half (220, 50.3%) of the respondents felt that the NCS tool allows for acceptance of staff inputs, whilst less than half (217, 49.7%) felt that the NCS tool has promoted the use of staff inputs to identify relevant innovations.

To determine the opinion of respondents on the contribution of NCS tool to their professional development, the following scores were obtained: the respondents believed that the NCS tool promotes continuous professional development (216, 49.2%), the sense of responsibility (205, 46.9%), the culture of learning (199, 45.5%), accountability (198, 45.3%) and mentoring system (193, 44.1%). Table 5 shows perceptions regarding lessons learnt from NCS.

**TABLE 5:** Perceptions of nurses regarding outcome standards of the National Core Standards tool.

Lesson learnt	Agree		Neutral		Disagree	
	*n*	%	*n*	%	*n*	%
The NCS promotes the use of feedback to improve service delivery	232	53.0	65	14.9	140	32.0
The NCS promotes the use of adverse events as a learning opportunity	225	51.4	71	16.2	141	32.2
The NCS allows for staff’s input	220	50.3	70	16.0	147	33.6
The NCS promotes the use of staff inputs to identify relevant innovations	217	49.7	74	16.9	146	33.4
The NCS promotes continuous professional developments	216	49.2	61	14.0	159	36.3
The NCS promotes culture of learning	199	45.5	75	17.2	163	37.2
The NCS promotes mentoring system	193	44.1	78	17.8	166	37.9
The NCS keeps the staff updated with relevant and current information	227	51.9	69	15.8	141	32.2

NCS, National Core Standards.

### Mean scores and standard deviation

The perceptions of professional nurses for using the NCS tool were measured using 28 items on an ordinal scale from ‘strongly agree’ to ‘strongly disagree’. For the 10 items used for structure of the NCS tool using the content of the tool as a construct, the mean score was 31.2 ± 9.9 (*α* = 0.8). To determine the mean score and standard deviation for the structure of the NCS tool using the organisational climate as a construct, three items were included, and the mean score was 6.7 ± 2.6 and Cronbach’s alpha was undetectable because there were too few items. To determine the ability of the NCS tool to ensure the elimination of wastage of available resources (process), including time, seven items were included, and the mean score was 21.6 ± 7.4 (*α* = 0.9). For the lessons learned from the NCS tool used to determine the outcomes, eight items were included, providing a mean score of 25.5 ± 7.8 (*α* = 0.8). Table 6 shows descriptive statistics and Cronbach’s alpha for these measurements.

**TABLE 6:** Descriptive statistics and Cronbach’s alpha.

Scale	Items	Mean	SD	Cronbach’s alpha
Structure of NCS	3	6.7	± 2.6	Undetectable
Process and contents of NCS tool	10	31.2	± 9.9	0.888
Process on elimination of waste	7	21.6	± 7.4	0.913
Outcome–lesson learnt	8	25.5	± 7.8	0.879

NCS, National Core Standards; SD, standard deviation.

## Discussion

### Nurses’ perceptions about the application of structure standards of the National Core Standards tool

Regarding the application of structure standards of the NCS tool, the highest level of agreement was for the availability of clear job descriptions in the organisation which enables application of the NCS tool. The other items were perceived by respondents as not offered by their organisations to enable the application of the NCS tool. These items had a below 50% level of agreement, with autonomy in decision-making being the lowest, scoring 38.6%.

The low rating of the practice environment poses a major concern because most authors have established a relationship between the nurse practice environment and health outcomes (Coetzee et al. 2013:163). Some authors believe that positive organisational climate is related to increased worker satisfaction (Castro & Martins 2010:2). Karam et al. (2018:71) stipulate that organisations are to create an environment that supports good interdisciplinary communication and collaboration between nurses and other healthcare workers.

### Nurses’ perceptions about the application of process standards of the National Core Standards tool

About 53% respondents felt that the standards put in NCS focused on patient-centred care. Patient-centredness means that the healthcare provider would respect and respond to patient’s needs, values and preferences, and the ethical decisions of health professionals would be guided by patient’s needs (Brand & Stiggelbout 2013:225). Effective implementation of patient-centredness requires motivated healthcare workers with a range of competencies and can partner with patients, families and other health workers (Bernabeo & Holmboe 2013:451). However, SAMA argued that the framework of accreditation offered by NCS is narrow, not covering all dimensions of quality as defined by the WHO, for instance, effectiveness, efficiency, accessibility and acceptability/patient-centredness (SAMA 2015:33).

Over half of the respondents (57.6%) believed that adherence to existing standards and clinical guidelines was part of staff performance criteria. This means that all respondents did not believe that adherence to the existing clinical guidelines and standards as provided in the NCS tool would improve staff performance. Kredo et al. (2017:1) asserted that presently there is no existing guidance or standard method in South Africa to efficiently and effectively develop and adapt clinical guidelines.

The results revealed that only 37.5% respondents believed that the implementation of NCS and its success to improve quality delivery was completely dependent on nurses, meaning a multidisciplinary approach is necessary. This means that most of the respondents believed that the implementation of NCS would be successful when other health professionals are involved in the process. Most authors believe that all healthcare professionals play an integral role in the coordination and delivery of quality healthcare (Balbale, Turcios & Lavela 2015:417; Bernabeo & Holmboe 2013:250). In order to provide high standard of healthcare and a better quality of life, quality initiatives must be engrained in the entire value chain of healthcare delivery (SAMA 2015:40)

This study reflected that 53.5% respondents believed that the NCS tool enabled them to identify areas of weakness, pointing to risks in basic human rights. Madisha (2015:25) mentioned that the purpose of developing NCS was to identify the health system’s strengths and gaps, to assess the current and future needs as well as the planning of future services, namely, planning for the implementation of National Health Insurance.

The analysis of the results revealed that 56.7% respondents believed that monitoring patient satisfaction surveys helped health establishments to determine the needs of their customers. Being direct recipients of care, patients are genuine assessors of quality (Izumi 2012:262). Patient satisfaction is defined as the extent to which patient’s expectations are met in the care provided. It depends on patient’s expectations, sex, age, education, type and stage of illness (Izumi 2012:263).

Most respondents felt that an independent body should do accreditation of health establishments to ensure credibility of findings. According to Standards Council of Canada, accreditation bodies should perform their work independently (ECONEX 2010:4). Independence is the primary purpose of accreditation to eradicate the presence of biased assessment that is likely to compromise accuracy. The question is the objectivity of the South African Office of Health Standard Compliance, it being government’s fully funded accreditation body (ECONEX 2010:4).

About 57.4% respondents believed that the NCS tool emphasises the multidisciplinary approach and the need for harmony at the workplace. According to Babiker et al. (2014:9), the best tool for constructing a more effective patient-centred healthcare delivery system, recognised globally, is by using an effective teamwork. In addition, Aiken et al. (2012:1717) declared that organisation must ruminate on nurses’ complaints as early warning signs for eroded quality care delivery, investigate these complaints and work on their solutions.

Evidence has emerged from data that most respondents did not believe that the NCS tool eliminates waste as suggested by the Lean system because of the low level of agreement, which was below 50%, with the ability to eliminate waste because of overproduction being at the lowest level, scoring just 43%.

### Nurses’ perceptions about outcome standards of the National Core Standards tool

This study revealed that about 53% respondents believed that the NCS tool promotes the use of feedback to improve delivery of service, and 51.4% of respondents believed that the tool promotes the use of adverse events as a learning opportunity. Percival et al. (2016:1) believed that any quality initiative could improve the quality of healthcare delivery by engaging frontline health practitioners through participatory feedback. The results showed low rating in the following: use of staff inputs to identify relevant innovations 217 (49.7%); promote continuous professional development 216 (49.2%); promote the culture of learning 199 (45.5%); and the lowest score being 193 (44.1%) for the response that the NCS tool has promoted the mentoring system. According to Mosadeghrad (2014:78), quality standards are more difficult *to establish* in service operations; therefore, organisations must invest in the continuous development of their employees to obtain positive outcomes in healthcare.

## Limitations of the study

Data collection was limited to professional nurses at few selected hospitals in KwaZulu-Natal. The study was confined to just one province of South Africa. Therefore, the results cannot be generalised to other provinces. As the NCS tool is used only in public institutions of South Africa, the results cannot be generalised for private institutions.

## Conclusion

In conclusion, a survey design was used to elicit nurses’ perceptions of NCS as a tool to improve the quality of healthcare delivery in public hospitals in KwaZulu-Natal tertiary hospitals. The findings of this study are as follows:

- Respondents disagreed that implementation of NCS and its success is completely dependent on nurses, meaning multidisciplinary approach is necessary. Currently, NCS is implemented, monitored and evaluated only by the nursing staff. Failure to involve other categories of healthcare workers leads to the misconception that NCS is only for nurses.
- Respondents believed that NCS enabled them to identify areas of weakness, pointing to risks to basic human rights. Less than half of the nurses believed that the NCS tool allowed staff inputs to identify relevant innovations. The NCS tool is structured into seven cross-cutting domains, whereas domain is defined as an area where quality and safety might be at risk (WHO 2006:8). Users’ inputs must be considered to ensure proper implementation of this tool.
- Respondents felt that the standards put in NCS focused on patient-centred care. According to De Jager and Du Plooy (2011:421), it is vital to implement health programmes that are patient-centred.
- Respondents believed that monitoring patient satisfaction surveys helps health establishments to determine the needs of their customers. Patient satisfaction is considered an important service quality indicator (Lyu et al. 2013:362). Several authors believed that knowing patients’ views about delivered healthcare is important because satisfied patients tend to adhere to treatment as well as treatment guidelines (Manary et al. 2013:201; Peltzer 2009:117).
- Most respondents did not believe that the NCS tool has an ability to ensure elimination of waste as suggested by the Lean system of quality care. The first step towards waste reduction is to identify value-added steps in every process, identify the value stream by providing value-added activities and eliminate everything which does not generate value to the product (Aziz & Hafez 2013:680; Kimsey 2010:53).

## Recommendations

- According to Ngidi and Dorasamy (2013:34), it is an arduous challenge to achieve lasting quality-improvement system in healthcare. The government of South Africa has a challenge to ensure that implementation of NCS involves all categories of workers employed in a healthcare setting. A quality coordinator must be employed in each area to oversee training and implementation of NCS by all healthcare workers.
- Standardised training, especially during orientation and induction programmes, should be instituted.
- In order to achieve the best possible results using available resources, any process undertaken should add value in terms of patient care. If it does not add measurable value, it is a waste and should be discontinued. Health information technology (computerised charting) should be instituted to have a link between all public hospitals so that all institutions could view a patient’s chart and avoid duplication. Waste represents misuse of resources; therefore, it must be reduced through education and training.
